# Evaluating the burden of occupational asthma among individuals aged 15 and older in India: A comprehensive study on mortality and disability-adjusted life years

**DOI:** 10.1016/j.pmedr.2025.103023

**Published:** 2025-02-26

**Authors:** Li Wei, Xiaoling Liu, Junhang Zhang, Donglei Shi, Zhaojun Wang

**Affiliations:** a Department of Thoracic Surgery, The Seventh Affiliated Hospital, Sun Yat-Sen University, Shenzhen, China; b Medical Record Department, The Seventh Affiliated Hospital, Sun Yat-Sen University, Shenzhen, China

**Keywords:** Occupational asthma, India, Global burden of disease, Death, Disability-adjusted life years

## Abstract

**Objective:**

Occupational asthma has become one of the most common occupational lung diseases, and its burden in India is underestimated.

**Methods:**

This study analyzed mortality and disability-adjusted life years (DALYs) caused by occupational asthma in India from 1990 to 2021 using data from the global burden of disease 2021. Data were categorized by sex, region, and age group, with age-standardized mortality rates (ASMR) and age-standardized DALYs rates (ASDR) as primary indicators to compare trends over time and across regions.

**Results:**

According to Global Burden of Disease 2021 estimates, there were a total of 11,575.6 deaths in India attributed to occupational asthma (95 % uncertainty interval, 7949.1–22,361), representing a 7.5 % increase since 1990. However, ASMR and ASDR showed significant declines across all sexes compared to 1990, with reductions of 55.9 % and 54.8 %, respectively. Geographically, the highest burden of occupational asthma was observed in Uttar Pradesh, while Goa had the lowest. In terms of sex, the burden of mortality and disability was notably higher among males, particularly within the 15–64 age group. In 2021, the burden across all age groups was primarily driven by years of life lost.

**Conclusions:**

While the overall burden of mortality due to occupational asthma continues to rise in India, ASMR and ASDR have declined. However, certain regions and demographic groups, especially Uttar Pradesh and the male population, continue to bear a disproportionate share of the burden. These findings underscore the critical need for targeted prevention and control measures for occupational asthma in India.

## Highlights

• Occupational asthma deaths in India rose by 7.5 %, but age-standardized mortality fell by 55.9 %.
• Uttar Pradesh had the highest burden.
• Males and those aged 15–64 faced the greatest risk.
• Targeted interventions are needed for high-risk regions and populations.

## 1 Introduction

Asthma is a chronic inflammatory respiratory disease characterized by episodic symptoms such as difficulty breathing, coughing, and wheezing (Hashmi and Cataletto, 2024). It currently affects over 300 million people worldwide (Cormier and Lemière, 2020). Asthma remains a significant public health challenge, with its global burden continuing to rise. According to the Global Burden of Disease (GBD) study in 2019, there were an estimated 262.41 million prevalent cases globally (Wang et al., 2023), and in the same year, the global death toll was 461,070 (Cao et al., 2022). Additionally, asthma imposes a substantial economic burden; in the United States alone, the total societal cost of asthma in 2007 was estimated at $56 billion (Cao et al., 2022).

Occupational asthma is a type of asthma linked to work-related exposures and is one of the most significant risk factors for adult asthma. An estimated 15 % to 33 % of adult asthma cases are linked to occupational exposures (Lau and Tarlo, 2019). The British Thoracic Society has identified over 400 causes of occupational asthma in its guidelines (Barber et al., 2022). India has the highest number of asthma patients worldwide, accounting for approximately 12.9 % of all asthma cases, with asthma-related deaths making up 42.3 % of the global total (Salvi et al., 2022). A 2014 cross-sectional study on Indian silk mill workers found an occupational asthma prevalence of 20.83 % (Gowda et al., 2014). However, asthma management in India is already subpar (Salvi et al., 2015), worsened by the complexity of diagnosing occupational asthma and a lack of clinical awareness of work-related asthma (Lau and Tarlo, 2019). Therefore, a thorough understanding of the burden of occupational asthma in India is especially important.

The 2021 GBD database is an extensive, complex scientific study that aims to quantify health losses caused by various diseases, injuries, and risk factors (Zhang et al., 2024). Although numerous GBD studies have reported on the burden of asthma in India, no specific studies have addressed the burden of occupational asthma in the country (India State-Level Disease Burden Initiative CRD Collaborators, 2018). This study uses the GBD database to analyze the overall burden of occupational asthma in India from 1990 to 2021, including trends across age groups. Our research aims to provide a basis for improved control of occupational asthma in India, supporting the development of effective strategies and policies.

## 2 Materials and methods

### 2.1 Overview

The latest GBD database for 2021 uses a standardized methodology to thoroughly evaluate the burden of 369 diseases, injuries, and risk factors across 204 countries and regions from 1990 to 2021. Detailed methods are available in numerous published articles (Ferrari et al., 2024; GBD 2021 Risk factors collaborators, 2024). All GBD risk factors are divided into four levels, with “occupational asthmagens” categorized under the fourth level within occupational risks. Because our study used publicly available data, ethical approval was not required.

Since GBD 2021 does not include data on occupational asthmagens for individuals under 15, this study only includes data for people aged 15 and older. All raw data were obtained from the Global Health Data Exchange website (https://vizhub.healthdata.org/gbd-results/). To assess the burden of occupational asthmagens in India, we utilized four indicators: mortality, disability-adjusted life years (DALYs), years of life lost (YLLs), and years lived with disability (YLDs). The specific data download process involved selecting “risk factor” for GBD estimate, “deaths, DALYs” for measure, “number and rate” for metrics, “occupational asthmagens” for risk, “asthma” for cause, “both, male, and female” for sex, and “1990–2021” for years. All data include a 95 % uncertainty interval. Additionally, the age-standardized rates provided by GBD 2021 are calculated per 100,000 population (Lai et al., 2024).

### 2.2 Definitions and risk estimation

Asthma is defined as a condition diagnosed by a physician within the past year, with patients experiencing persistent wheezing. Relevant international classification of diseases-10 codes include J45 and J46, while the international classification of diseases-9 code is 493. Occupational asthmagens are defined as the proportion of asthma cases among workers in nine occupational categories: legislators, senior officials, and managers; professionals; technicians and associate professionals; clerks; service workers and shop/market sales workers; skilled agricultural and fishery workers; plant and machine operators and assemblers; craft and related workers; and elementary occupations (GBD 2021 Risk factors collaborators, 2024).

The initial data were obtained from the International Labor Organization, and the analysis was performed using the comparative risk assessment framework, which mainly consists of seven methodological steps. First, data were synthesized through a systematic review of risk-outcome pairs based on the methods and evaluation criteria of the World Cancer Research Fund. Second, exposure data were collected, and exposure levels and distributions were estimated using two Bayesian statistical models (spatiotemporal Gaussian process regression and disease modeling meta-regression 2.1). Third, the theoretical minimum risk exposure levels were determined as the exposure levels associated with the lowest risk of adverse health outcomes. Fourth, population attributable fractions were calculated for each risk-outcome pair to quantify the proportion of disease burden attributable to specific risk factors. Fifth, age-specific risk-weighted exposure prevalence was estimated for each risk factor. Sixth, mediating factors were incorporated to correct for potential overestimation of population attributable fractions. Lastly, attributable burden estimates were calculated for each combination of age group, sex, location, and year. The following equation was used to determine the exposure rate of occupational asthma:

$${\mathrm{Exposure}}_{c,y,s,a,r}=\sum_{\mathrm{EA}}{\mathrm{Proportion}}_{\mathrm{occ},c,y}*{\mathrm{EAP}}_{c,y,s,a}$$

EAP = economically active population, c = country, r = risk, EA = economic activity, a = age, s = sex, OCC = occupation, y = year (GBD 2021 Risk factors collaborators, 2024).

### 2.3 Statistical analysis

We examined the overall and sex-specific burden trends of occupational asthma in India from 1990 to 2021. We further analyzed the burden across Indian states for the year 2021, calculating changes relative to 1990. Additionally, we evaluated the burden across different age groups in 2021. To calculate changes from 1990 to 2021, we used a specified formula. $\text{Percent change}\;\left(\%\right)=\frac{\text{Data in}\;2021‐\text{Data in}\;1990}{\text{Data in}\;1990}\times 100\%$. Data analysis and visualization were conducted using R statistical software (version 4.3.2) and the “ggplot2” package.

## 3 Results

### 3.1 The burden of occupational asthma in India

In 2021, India recorded a total of 11,575.6 deaths (95 % uncertainty interval: 7949.1–22,361) attributed to occupational asthma, representing a 7.5 % increase from 1990. This included 2575.7 deaths among females and 8999.9 deaths among males, reflecting increases of 11.5 % and 6.4 %, respectively, compared to 1990. The age-standardized mortality rates (ASMR) in 2021 for both sexes, males, and females were 0.9 (95 % uncertainty interval: 0.62–1.75), 1.43 (95 % uncertainty interval: 0.91–3.17), and 0.39 (95 % uncertainty interval: 0.21–0.69), respectively, each showing significant declines from 1990, with reductions of 55.9 %, 54.2 %, and 55.7 % (Table 1). Similarly, the age-standardized DALYs rates (ASDR) for both sexes, males, and females in 2021 were 33.18 (95 % uncertainty interval, 24.12–57.42), 51.85 (95 % uncertainty interval, 35.99–100.39), and 14.82 (95 % uncertainty interval, 8.66–23.86), respectively, with decreases of 54.8 %, 52.8 %, and 52.8 % compared to 1990 (Table 2).

**Table 1:** Number of deaths and age-standardized mortality rates for occupational asthma among individuals aged 15 and older in India, 1990–2021.

location	sex	Number 1990	Number 2021	Percentage change	ASMR 1990	ASMR 2021	Percentage change
India	Both	10,772.2 (7082.9-19,231.1)	11,575.6 (7949.1-22,361)	7.5 %	2.04 (1.29–3.83)	0.9 (0.62–1.75)	−55.9 %
	Female	2310.4 (1263.3-3856.8)	2575.7 (1382.2-4403.2)	11.5 %	0.88 (0.47–1.53)	0.39 (0.21–0.69)	−55.7 %
	Male	8461.8 (5014.8-17,372.4)	8999.9 (5854.8-19,504.4)	6.4 %	3.12 (1.76–6.77)	1.43 (0.91–3.17)	−54.2 %
Andhra Pradesh	Both	454.1 (284.1–714.4)	330.3 (207.6–502.6)	−27.3 %	1.66 (1.02–2.73)	0.56 (0.35–0.85)	−66.3 %
	Female	157.8 (79–281.4)	126.9 (58–261.7)	−19.6 %	1.16 (0.56–2.17)	0.41 (0.19–0.84)	−64.7 %
	Male	296.2 (152.6–569)	203.4 (115.3–346.8)	−31.3 %	2.16 (1.07–4.31)	0.72 (0.41–1.24)	−66.7 %
Arunachal Pradesh	Both	6.5 (4.1–10.3)	5.8 (3.7–8.8)	−10.8 %	1.48 (0.89–2.42)	0.55 (0.35–0.85)	−62.8 %
	Female	1.5 (0.7–2.9)	1.5 (0.7–3)	0	0.73 (0.35–1.51)	0.28 (0.12–0.56)	−61.6 %
	Male	5.1 (2.9–8.6)	4.3 (2.6–6.7)	−15.7 %	2.09 (1.1–3.79)	0.8 (0.46–1.26)	−61.7 %
Assam	Both	187.1 (111.8–322.8)	186 (111–316.6)	−0.6 %	1.53 (0.87–2.79)	0.64 (0.36–1.13)	−58.2 %
	Female	31 (16–52)	24.9 (11.2–49)	−19.7 %	0.48 (0.24–0.82)	0.15 (0.07–0.3)	−68.7 %
	Male	156.1 (86.3–287.2)	161.1 (91.4–290.5)	3.2 %	2.39 (1.22–4.66)	1.12 (0.6–2.1)	−53.1 %
Bihar	Both	738.7 (401.1–1739.5)	557.7 (309–1467.5)	−24.5 %	1.96 (1.02–4.83)	0.62 (0.34–1.62)	−68.4 %
	Female	117.5 (47.9–244.4)	78.3 (29.4–173.4)	−33.4 %	0.64 (0.24–1.35)	0.18 (0.07–0.4)	−71.9 %
	Male	621.3 (300.6–1664.8)	479.3 (237.7–1397)	−22.9 %	3.14 (1.43–8.83)	1.03 (0.51–2.99)	−67.2 %
Chhattisgarh	Both	198.2 (123–318.7)	270.5 (154–456.8)	36.5 %	1.91 (1.17–3.27)	1.01 (0.56–1.79)	−47.1 %
	Female	66.3 (32.7–116.4)	90 (34.4–180.5)	35.7 %	1.2 (0.57–2.23)	0.62 (0.23–1.25)	−48.3 %
	Male	131.9 (70.1–251.4)	180.4 (90.2–346)	36.8 %	2.76 (1.34–5.7)	1.45 (0.7–2.89)	−47.5 %
Delhi	Both	51.5 (27.9–113.4)	52.1 (35.3–108.2)	1.2 %	0.99 (0.51–2.3)	0.27 (0.18–0.57)	−72.7 %
	Female	3.5 (1.7–5.8)	4.1 (2–6.8)	17.1 %	0.12 (0.06–0.22)	0.04 (0.02–0.07)	−66.7 %
	Male	48 (25–110.8)	48.1 (31–103.1)	0.2 %	1.68 (0.84–4.02)	0.5 (0.32–1.09)	−70.2 %
Goa	Both	2.1 (1.3–3.2)	1.6 (1.1–2.3)	−23.8 %	0.22 (0.14–0.35)	0.08 (0.05–0.11)	−63.6 %
	Female	0.6 (0.2–1.2)	0.4 (0.2–0.8)	−33.3 %	0.11 (0.05–0.24)	0.04 (0.02–0.07)	−63.6 %
	Male	1.5 (0.9–2.6)	1.2 (0.7–1.8)	−20 %	0.33 (0.19–0.58)	0.12 (0.08–0.18)	−63.6 %
Gujarat	Both	495.4 (311.7–853.5)	631.3 (410.5–1001)	27.4 %	1.87 (1.13–3.48)	0.93 (0.6–1.51)	−50.3 %
	Female	91.3 (45.6–164.9)	110.5 (52.5–216.3)	21 %	0.64 (0.31–1.2)	0.31 (0.15–0.61)	−51.6 %
	Male	404.1 (236.8–780.7)	520.9 (327.4–932.9)	28.9 %	3.14 (1.7–6.48)	1.6 (0.98–2.92)	−49 %
Haryana	Both	147 (89.8–295.8)	163.7 (101.7–331.2)	11.4 %	1.46 (0.87–3.05)	0.61 (0.37–1.25)	−58.2 %
	Female	15.8 (7.8–29.6)	20 (10.3–38.6)	26.6 %	0.32 (0.15–0.62)	0.14 (0.07–0.28)	−56.2 %
	Male	131.2 (76.7–284)	143.7 (83.4–311.8)	9.5 %	2.42 (1.37–5.42)	1.07 (0.62–2.37)	−55.8 %
Himachal Pradesh	Both	100.6 (64.9–170.2)	102.3 (64.7–156)	1.7 %	2.91 (1.84–5.01)	1.21 (0.76–1.85)	−58.4 %
	Female	33.9 (15.3–63)	33.4 (14.3–69)	−1.5 %	2.01 (0.89–3.83)	0.75 (0.32–1.54)	−62.7 %
	Male	66.7 (35.8–133.7)	68.9 (42.9–102.3)	3.3 %	3.69 (1.91–7.49)	1.73 (1.08–2.56)	−53.1 %
Jammu and Kashmir	Both	100.9 (59.4–212.8)	94.9 (60.2–162.4)	−5.9 %	2.47 (1.37–5.37)	0.83 (0.52–1.42)	−66.4 %
	Female	5.6 (2.4–11.4)	5.4 (2.6–10.1)	−3.6 %	0.3 (0.11–0.72)	0.09 (0.04–0.18)	−70 %
	Male	95.3 (55–207.9)	89.5 (54.1–155.8)	−6.1 %	4.16 (2.24–9.26)	1.53 (0.93–2.67)	−63.2 %
Jharkhand	Both	268.5 (153.3–514.1)	137 (74.4–259.4)	−49 %	2.11 (1.15–4.27)	0.44 (0.23–0.85)	−79.1 %
	Female	69 (28.9–143.8)	67.1 (23.3–173)	−2.8 %	1.13 (0.45–2.53)	0.45 (0.15–1.17)	−60.2 %
	Male	199.5 (93.2–452.2)	69.9 (38.5–153.7)	−65 %	2.99 (1.31–7.23)	0.44 (0.24–0.97)	−85.3 %
Karnataka	Both	644.2 (403.8–1026.9)	583.3 (378.7–910.6)	−9.5 %	2.23 (1.33–3.66)	0.86 (0.55–1.35)	−61.4 %
	Female	171.9 (85.9–316.2)	169 (80.8–322.2)	−1.7 %	1.15 (0.55–2.23)	0.48 (0.23–0.92)	−58.3 %
	Male	472.3 (270.6–838.8)	414.4 (256–677.6)	−12.3 %	3.31 (1.78–6.21)	1.28 (0.78–2.13)	−61.3 %
Kerala	Both	193.6 (117.7–302.9)	97.6 (69.6–134.1)	−49.6 %	0.9 (0.53–1.42)	0.21 (0.15–0.28)	−76.7 %
	Female	28 (12.4–60.4)	25.9 (14.2–47.4)	−7.5 %	0.24 (0.1–0.54)	0.1 (0.06–0.18)	−58.3 %
	Male	165.6 (91.8–280.5)	71.7 (49.5–105)	−56.7 %	1.64 (0.88–2.79)	0.33 (0.23–0.49)	−79.9 %
Madhya Pradesh	Both	712 (419.9–1335.6)	881.6 (511.4–1612.5)	23.8 %	2.43 (1.37–4.74)	1.19 (0.68–2.22)	−51 %
	Female	162 (76.2–292.7)	181.3 (71.8–385.5)	11.9 %	1.12 (0.5–2.14)	0.48 (0.19–1.03)	−57.1 %
	Male	550 (284.3–1168.5)	700.3 (397.6–1405.3)	27.3 %	3.66 (1.81–8.36)	1.96 (1.08–3.99)	−46.4 %
Maharashtra	Both	737 (463.5–1161.3)	710 (444.4–1095.1)	−3.7 %	1.49 (0.89–2.43)	0.56 (0.35–0.87)	−62.4 %
	Female	282.4 (131.6–544.9)	321 (140.7–602.9)	13.7 %	1.17 (0.51–2.4)	0.5 (0.22–0.93)	−57.3 %
	Male	454.6 (265.2–799.5)	389 (244.7–644)	−14.4 %	1.81 (0.99–3.4)	0.63 (0.39–1.04)	−65.2 %
Manipur	Both	10.5 (6.2–17.6)	14.8 (9.1–21.9)	41 %	1 (0.57–1.84)	0.5 (0.3–0.75)	−50 %
	Female	3.2 (1.6–6.3)	6.1 (3.2–10.8)	90.6 %	0.61 (0.29–1.25)	0.39 (0.2–0.7)	−36.1 %
	Male	7.3 (3.8–14.9)	8.7 (4.3–14.6)	19.2 %	1.37 (0.65–2.94)	0.61 (0.29–1.02)	−55.5 %
Meghalaya	Both	9.7 (6–14.9)	13.7 (8.4–20.7)	41.2 %	1.21 (0.72–1.97)	0.64 (0.38–0.96)	−47.1 %
	Female	2.6 (1.3–5.6)	4.7 (2.2–10.8)	80.8 %	0.65 (0.31–1.44)	0.41 (0.18–0.92)	−36.9 %
	Male	7.1 (3.7–12)	8.9 (5.3–14.7)	25.4 %	1.7 (0.86–3.06)	0.89 (0.5–1.5)	−47.6 %
Mizoram	Both	8.7 (5.6–13.2)	11.9 (8.1–17.7)	36.8 %	2.53 (1.57–3.98)	1.16 (0.78–1.74)	−54.2 %
	Female	3.2 (1.6–5.9)	4.7 (2.2–8.9)	46.9 %	1.98 (0.94–3.83)	0.9 (0.42–1.69)	−54.5 %
	Male	5.5 (3.2–9.9)	7.2 (4.5–11.3)	30.9 %	3.04 (1.7–5.71)	1.43 (0.88–2.25)	−53 %
Nagaland	Both	3.3 (2–5.6)	2.9 (1.7–4.5)	−12.1 %	0.49 (0.29–0.89)	0.21 (0.12–0.34)	−57.1 %
	Female	0.8 (0.3–1.7)	1 (0.4–2.4)	25 %	0.29 (0.11–0.64)	0.16 (0.06–0.38)	−44.8 %
	Male	2.5 (1.4–4.5)	1.9 (1.1–3.2)	−24 %	0.65 (0.35–1.24)	0.26 (0.15–0.44)	−60 %
Odisha	Both	138.6 (79.2–229.7)	142.6 (82.8–252.6)	2.9 %	0.64 (0.35–1.11)	0.29 (0.17–0.52)	−54.7 %
	Female	25.9 (11.4–69.6)	24.7 (9.5–60.2)	−4.6 %	0.23 (0.1–0.61)	0.1 (0.04–0.24)	−56.5 %
	Male	112.6 (57.5–206.3)	117.9 (66–213.7)	4.7 %	1.06 (0.51–2.05)	0.49 (0.27–0.9)	−53.8 %
Punjab	Both	87.7 (50.8–181.5)	86.2 (55–145.2)	−1.7 %	0.64 (0.36–1.39)	0.25 (0.16–0.43)	−60.9 %
	Female	8.1 (3.6–16.7)	11.7 (5.7–25.2)	44.4 %	0.13 (0.05–0.29)	0.07 (0.03–0.15)	−46.2 %
	Male	79.6 (43.8–170.9)	74.6 (45.1–132.3)	−6.3 %	1.07 (0.57–2.44)	0.44 (0.26–0.77)	−58.9 %
Rajasthan	Both	941.9 (568.2–1794.8)	1172.7 (707.1–2034.5)	24.5 %	3.69 (2.16–7.36)	1.83 (1.1–3.2)	−50.4 %
	Female	170.5 (72.6–332.9)	250.9 (98.5–500.9)	47.2 %	1.33 (0.57–2.73)	0.75 (0.29–1.5)	−43.6 %
	Male	771.4 (439.1–1639.3)	921.7 (517.7–1672.1)	19.5 %	5.92 (3.19–13.05)	3.04 (1.69–5.53)	−48.6 %
Sikkim	Both	3.9 (2.5–6)	3.1 (2–4.9)	−20.5 %	2.02 (1.27–3.29)	0.54 (0.34–0.83)	−73.3 %
	Female	1.3 (0.6–2.3)	1.2 (0.5–2.4)	−7.7 %	1.47 (0.69–2.8)	0.43 (0.19–0.91)	−70.7 %
	Male	2.6 (1.4–4.6)	1.9 (1.2–3.2)	−26.9 %	2.45 (1.3–4.69)	0.63 (0.37–1.04)	−74.3 %
Tamil Nadu	Both	545.1 (354.2–827.8)	367.3 (243.7–550)	−32.6 %	1.31 (0.83–2.07)	0.4 (0.26–0.59)	−69.5 %
	Female	189.7 (94.4–340.1)	148.9 (72–299.2)	−21.5 %	0.92 (0.44–1.7)	0.31 (0.15–0.62)	−66.3 %
	Male	355.4 (196.7–612.4)	218.4 (142.2–319.3)	−38.5 %	1.67 (0.87–3.07)	0.49 (0.32–0.72)	−70.7 %
Telangana	Both	372.6 (236.6–590.4)	287.3 (177.9–455)	−22.9 %	2.17 (1.34–3.49)	0.76 (0.47–1.21)	−65 %
	Female	145.7 (72.7–254.7)	125.8 (57.3–237.2)	−13.7 %	1.71 (0.81–3.06)	0.63 (0.29–1.19)	−63.2 %
	Male	227 (122.3–439.5)	161.5 (96.1–264.6)	−28.9 %	2.62 (1.36–5.22)	0.9 (0.52–1.47)	−65.6 %
Tripura	Both	31 (18.2–52.1)	25.3 (16.1–38.6)	−18.4 %	2 (1.12–3.45)	0.66 (0.41–1)	−67 %
	Female	4.1 (2–7.6)	3.5 (1.7–7.4)	−14.6 %	0.5 (0.24–0.95)	0.17 (0.08–0.36)	−66 %
	Male	26.8 (14.7–47.1)	21.8 (13.4–34.6)	−18.7 %	3.38 (1.74–6.21)	1.15 (0.7–1.84)	−66 %
Union Territories other than Delhi	Both	7.6 (4.8–12.3)	10.6 (6.8–16.8)	39.5 %	0.64 (0.38–1.06)	0.28 (0.18–0.45)	−56.2 %
	Female	2.4 (1.1–4.6)	3.6 (1.7–7.2)	50 %	0.41 (0.17–0.82)	0.19 (0.09–0.39)	−53.7 %
	Male	5.2 (3–9.4)	7 (4.4–11.1)	34.6 %	0.86 (0.44–1.6)	0.37 (0.23–0.6)	−57 %
Uttar Pradesh	Both	2784.5 (1666.2-6060.5)	3925.8 (2357.9-9866.2)	41 %	3.47 (1.98–8.01)	2.14 (1.25–5.42)	−38.3 %
	Female	393.4 (174.8–739.1)	601.6 (245.6–1187.3)	52.9 %	1.05 (0.46–2.03)	0.65 (0.26–1.29)	−38.1 %
	Male	2391.1 (1299.6-5723.4)	3324.2 (1813-9557.5)	39 %	5.51 (2.86–14.02)	3.63 (1.94–10.55)	−34.1 %
Uttarakhand	Both	177.9 (108.6–280.2)	209.3 (120–333.1)	17.7 %	4.52 (2.69–7.27)	1.94 (1.09–3.11)	−57.1 %
	Female	45.4 (20.3–82)	52.4 (21.4–110.5)	15.4 %	2.24 (1–4.24)	0.93 (0.37–1.93)	−58.5 %
	Male	132.5 (71.6–219.2)	156.9 (90.9–249.4)	18.4 %	6.93 (3.53–12.08)	3.09 (1.76–4.98)	−55.4 %
West Bengal	Both	611.9 (375.9–1091.6)	496.2 (310.6–891.8)	−18.9 %	1.57 (0.89–2.92)	0.5 (0.3–0.88)	−68.2 %
	Female	76.4 (38.9–134.3)	75 (39.6–138.2)	−1.8 %	0.37 (0.18–0.7)	0.15 (0.08–0.27)	−59.5 %
	Male	535.5 (297.6–1017.7)	421.2 (246–793.2)	−21.3 %	2.68 (1.37–5.35)	0.83 (0.47–1.52)	−69 %

Note: ASMR, age-standardized mortality rates.

**Table 2:** Number of DALYs and age-standardized DALYs rates for occupational asthma among individuals aged 15 and older in India, 1990–2021.

location	sex	Number 1990	Number 2021	Percentage change	ASDR 1990	ASDR 2021	Percentage change
India	Both	433,720.4 (307,528.8-692,883)	445,982.6 (325,561.7-761,427.5)	2.8 %	73.45 (50.74–122.51)	33.18 (24.12–57.42)	−54.8 %
	Female	98,285.7 (58,862.3-151,219.8)	100,357.2 (59,038.7-160,292.6)	2.1 %	33.8 (19.84–53.55)	14.82 (8.66–23.86)	−56.2 %
	Male	335,434.7 (223,135.3-591,524.3)	345,625.4 (242,038.2-653,962.1)	3 %	109.87 (71.15–207.28)	51.85 (35.99–100.39)	−52.8 %
Andhra Pradesh	Both	19,377.2 (13,326.7-27,935.2)	13,273.1 (9129.7-19,061.8)	−31.5 %	63.44 (42.77–93.15)	22.19 (15.26–31.9)	−65 %
	Female	6898.8 (3917.4-11,435.5)	5044.6 (2581.1-9074.1)	−26.9 %	45.52 (25.06–76.87)	16.31 (8.33–29.58)	−64.2 %
	Male	12,478.4 (7567.6-21,154.2)	8228.5 (5389.3-12,509.9)	−34.1 %	80.9 (48.15–143.82)	28.61 (18.67–43.7)	−64.6 %
Arunachal Pradesh	Both	292.9 (203.1–410.1)	264.5 (186.6–368.1)	−9.7 %	55.6 (37.42–82.58)	21.11 (14.52–29.7)	−62 %
	Female	70.7 (41.2–121.1)	72.1 (37.1–131.7)	2 %	29.66 (16.72–53.23)	11.44 (5.69–21.17)	−61.4 %
	Male	222.3 (144.9–342.4)	192.4 (130.8–273.6)	−13.5 %	75.56 (46.61–120.65)	29.97 (19.56–43.60)	−60.3 %
Assam	Both	8324.7 (5617.3-12,526.8)	8155.7 (5452-12,263.9)	−2 %	59.15 (38.56–94.69)	25.34 (16.56–39.04)	−57.2 %
	Female	1640.2 (943–2558.7)	1255.2 (642–2273)	−23.5 %	22.86 (12.79–37.02)	7.32 (3.70–13.65)	−68 %
	Male	6684.5 (4311.3-10,643.5)	6900.6 (4432.6-10,727.1)	3.2 %	88.77 (53.77–150.2)	42.97 (27.59–69.65)	−51.6 %
Bihar	Both	27,849 (17,113.9-56,500)	20,249.3 (12,697.6-45,882.8)	−27.3 %	66.74 (39.87–142.5)	21.2 (13.13–49.26)	−68.2 %
	Female	4444.3 (2067.6-8445.7)	2850.3 (1255.2-5805.5)	−35.9 %	22.32 (10.26–43.98)	6.19 (2.65–12.80)	−72.3 %
	Male	23,404.7 (13,338.5-52,565)	17,399.1 (10,181.3-43,902.3)	−25.7 %	106.09 (58.79–252.23)	35.4 (20.32–91.89)	−66.6 %
Chhattisgarh	Both	8250.1 (5697.7-12,000.6)	10,994.9 (6898.7-16,955.7)	33.3 %	70.88 (47.35–106.46)	37.49 (22.99–59.21)	−47.1 %
	Female	2930.2 (1655.6-4631.5)	3858.2 (1668.6-7199.5)	31.7 %	48.5 (26.37–78.64)	25.22 (10.94–48.09)	−48 %
	Male	5319.9 (3192.4-9056.4)	7136.7 (3914.6-12,322)	34.2 %	95.61 (53.90–174.22)	50.93 (27.26–90.46)	−46.7 %
Delhi	Both	2504 (1629-4681.7)	2536.2 (1825.9-4513.3)	1.3 %	40.11 (24.13–81.53)	12.47 (8.91–22.42)	−68.9 %
	Female	205.2 (118.2–313.7)	220.2 (133.9–340.2)	7.3 %	6.33 (3.55–9.81)	2.15 (1.29–3.32)	−66 %
	Male	2298.9 (1456.3-4476.4)	2316.1 (1637.4-4256.9)	0.7 %	66.57 (39.33–141.63)	22.46 (15.89–41.52)	−66.3 %
Goa	Both	123.2 (85.8–167.2)	91 (66.3–120.9)	−26.1 %	11.59 (7.88–15.92)	4.72 (3.43–6.22)	−59.3 %
	Female	32.6 (18–56.3)	22 (12.8–36.4)	−32.5 %	6.01 (3.2–10.75)	2.26 (1.31–3.65)	−62.4 %
	Male	90.6 (61–134.8)	68.9 (48.4–93.8)	−24 %	17.18 (11.19–26.02)	7.24 (5.11–9.85)	−57.9 %
Gujarat	Both	21,417.6 (15,196.6-32,367.6)	25,373.6 (18,171.5-37,206.4)	18.5 %	71.96 (48.72–115.46)	35.76 (25.26–53.08)	−50.3 %
	Female	4462.8 (2568.6-7245.8)	4913.3 (2505.9-8943.6)	10.1 %	28.76 (15.77–48.98)	13.72 (6.97–25.05)	−52.3 %
	Male	16,954.8 (11,117.8-28,431)	20,460.2 (13,989.7-32,951.7)	20.7 %	114.52 (72.61–204.01)	58.33 (39.31–96.04)	−49.1 %
Haryana	Both	6227.8 (4302.4-10,786.7)	6903.5 (4709.8-12,106.3)	10.8 %	56.27 (38.07–101.6)	24.21 (16.24–43.36)	−57 %
	Female	811.4 (462.1–1342.5)	956.4 (539.5–1746.6)	17.9 %	15.02 (8.27–25.55)	6.67 (3.7–12.18)	−55.6 %
	Male	5416.3 (3614.4-10,157.3)	5947.1 (3934.3-11,125.9)	9.8 %	91.74 (59.66–178.46)	41.47 (26.85–78.71)	−54.8 %
Himachal Pradesh	Both	3751.1 (2564.1-5697.1)	3620.5 (2488.6-5357.8)	−3.5 %	99.67 (67.66–157.31)	41.6 (28.45–61.77)	−58.3 %
	Female	1355.4 (729.5–2222.7)	1165.8 (555.6–2269.9)	−14 %	72.87 (38.30–123.68)	25.85(12.32–50.27)	−64.5 %
	Male	2395.7 (1494.8-4413.9)	2454.7 (1689.8-3534.9)	2.5 %	123.8 (75.56–231.5)	58.72(40.37–83.42)	−52.6 %
Jammu and Kashmir	Both	3879.6 (2530.9-7047.9)	3645.1 (2501.6-5646.2)	−6 %	81.67 (50.25–161.49)	29.69(19.99–46.43)	−63.6 %
	Female	258.2 (137.8–452.2)	227.7 (125.4–380.6)	−11.8 %	10.72 (5.24–21.06)	3.63 (1.93–6.2)	−66.1 %
	Male	3621.3 (2330.7-6772.4)	3417.4 (2338.1-5403.7)	−5.6 %	137.41 (83.66–278.73)	54.3 (36.09–86.74)	−60.5 %
Jharkhand	Both	10,268.5 (6442.9-17,526.1)	5288.2 (3249.3-8959.1)	−48.5 %	71.44 (44.02–128.42)	15.9 (9.64–27.3)	−77.7 %
	Female	2642.2 (1317.3-4711.2)	2357.3 (999.5–5543.4)	−10.8 %	38.34 (18.07–74.89)	14.7 (6.09–34.6)	−61.7 %
	Male	7626.3 (4325.7-15,047.4)	2931 (1846.8-5516.5)	−61.6 %	101.03 (54.82–213.11)	17.13 (10.62–32.27)	−83 %
Karnataka	Both	26,235.4 (18,126.0-37,860.1)	22,761.8 (15,945.3-32,332.3)	−13.2 %	81.18 (54.33–122.65)	32.12 (22.32–46.05)	−60.4 %
	Female	7622.1 (4273.6-12,531.5)	6866.3 (3688.7-12,420.3)	−9.9 %	46.28 (24.76–79.53)	18.9 (10.15–34.46)	−59.2 %
	Male	18,613.3 (11,705-29,763.9)	15,895.5 (10,505.1-23,799.8)	−14.6 %	115.1 (69.99–192.67)	46.29 (30.5–70.29)	−59.8 %
Kerala	Both	7922.3 (5381.6-11,441.6)	4153.7 (3149.4-5431.5)	−47.6 %	33.53 (22.25–48.85)	9.15 (6.96–11.81)	−72.7 %
	Female	1342.2 (737.5–2464.7)	1066.4 (663.0–1774.8)	−20.5 %	10.56 (5.65–20.12)	4.39 (2.77–7.18)	−58.4 %
	Male	6580.1 (4300.7-10,251.7)	3087.3 (2294.9-4100.9)	−53.1 %	58.58 (36.71–92.71)	14.73 (10.92–19.5)	−74.9 %
Madhya Pradesh	Both	28,745.3 (19,050.2-47,209)	34,044.5 (21,724.7-55,768.8)	18.4 %	87.42 (55.57–149.78)	43.14 (27.14–71.7)	−50.7 %
	Female	6989.4 (3860.1-11,359.1)	7188.2 (3322.3-14,070.2)	2.8 %	42.99 (23.14–72.02)	17.88 (8.07–35.16)	−58.4 %
	Male	21,755.9 (12,882.6-40,540.7)	26,856.3 (16,631.1-48,815.8)	23.4 %	128.05 (72.35–249.95)	69.13 (42.48–128.33)	−46 %
Maharashtra	Both	30,367.5 (21,346.6-42,533.1)	27,969.7(19,557.8-38,808)	−7.9 %	53.53 (35.91–77.98)	21.17 (14.69–29.6)	−60.5 %
	Female	11,243.6 (6120.4-19,154.7)	11,506.7 (5788.6-19,610.9)	2.3 %	40.97 (21.70–73.84)	17.41 (8.68–29.96)	−57.5 %
	Male	19,123.9 (12,923.6-29,789)	16,463 (11,635-24,375.4)	−13.9 %	65.5 (42.60–106.64)	24.91 (17.46–36.58)	−62 %
Manipur	Both	426.3 (283.9–628.4)	573.1 (391.4–799.7)	34.4 %	35.36 (22.67–54.52)	17.52 (11.79–24.55)	−50.5 %
	Female	135.7 (79.1–246.9)	233.5 (132.7–381.9)	72.1 %	22.65 (12.66–42.4)	13.87 (7.87–22.76)	−38.8 %
	Male	290.6 (180.8–504)	339.5 (205.7–533.3)	16.8 %	47.01 (27.49–85.18)	21.36 (12.59–33.84)	−54.6 %
Meghalaya	Both	451.1 (315.9–634.5)	603.9 (410.9–882.6)	33.9 %	45.43 (29.99–68.11)	24.08 (16.01–35.39)	−47 %
	Female	140.2 (85.4–248.9)	221.5 (119.8–424.1)	58 %	28.24 (16.17–54.9)	16.69 (8.74–33.19)	−40.9 %
	Male	310.9 (203.9–463.9)	382.4 (252.8–570.1)	23 %	60.16 (37.03–93.71)	32.17 (20.49–49.59)	−46.5 %
Mizoram	Both	372.1 (267.5–518.9)	465.1 (333.2–657.1)	25 %	90.72 (62.60–129.88)	41.66 (29.41–59.37)	−54.1 %
	Female	139.6 (79.5–228.2)	178.6 (92–313.6)	27.9 %	71.83 (39.78–121.23)	31.55 (16.11–55.99)	−56.1 %
	Male	232.5 (157.9–362.3)	286.5 (193.1–428.1)	23.2 %	107.59 (68.36–182.99)	52.26 (34.81–78.06)	−51.4 %
Nagaland	Both	167.6 (118.2–245.5)	142 (93.4–200.4)	−15.3 %	21.56 (14.83–33.09)	9.34 (6.04–13.4)	−56.7 %
	Female	39.6 (19.7–72.3)	42.9 (19.8–89.9)	8.3 %	11.71 (5.82–22.47)	6.04 (2.74–12.65)	−48.4 %
	Male	128 (85.3–192.7)	99.2 (65–144.2)	−22.5 %	29.08 (18.72–46.32)	12.37 (7.92–18.26)	−57.5 %
Odisha	Both	6455.5 (4216.3-9255.9)	6008.3 (3992-9472.1)	−6.9 %	26.68 (17.04–39.23)	12.03 (7.95–18.85)	−54.9 %
	Female	1381.3 (778–3110.6)	1135.7 (534.8–2260.1)	−17.8 %	11.09 (6.06–25.76)	4.52 (2.12–9.01)	−59.2 %
	Male	5074.1 (3108-7829.8)	4872.5 (3124.6-8102.1)	−4 %	41.98 (25.01–67.93)	19.65 (12.56–32.67)	−53.2 %
Punjab	Both	3949.8 (2620.3-6337.7)	3659.9 (2571.1-5301)	−7.3 %	25.58 (16.44–44.21)	10.48 (7.37–15.29)	−59 %
	Female	355 (191.8–631.3)	446.4 (256.6–806.2)	25.7 %	5.01 (2.63–9.32)	2.6 (1.48–4.72)	−48.1 %
	Male	3594.8 (2342.3-6010.8)	3213.5 (2221.8-4947)	−10.6 %	43.26 (27.61–76.91)	18.3 (12.59–28.37)	−57.7 %
Rajasthan	Both	36,969.7 (24,452.2-62,922.5)	45,606.2 (29,835.1-71,798.3)	23.4 %	130.11 (83.27–229.69)	66.42 (42.35–106.71)	−49 %
	Female	7316.1 (3730.9-12,943.9)	10,155.2 (4500.1-19,293)	38.8 %	51.74 (25.40–94.46)	28.66 (12.4–54.78)	−44.6 %
	Male	29,653.5 (19,257.7-55,504.5)	35,451.1 (21,253.5-56,771.4)	19.6 %	202.58 (125.91–402.59)	106.38 (61.23–176.21)	−47.5 %
Sikkim	Both	161.2 (114.4–223.5)	130.2 (90.2–184.8)	−19.2 %	69.34 (47.74–102.55)	20.15(13.84–28.88)	−70.9 %
	Female	54.9 (31.2–91.7)	48.8 (24.1–87.6)	−11.1 %	53.25 (29.63–91.44)	15.93 (7.74–28.95)	−70.1 %
	Male	106.3 (68.3–163.3)	81.5 (56.1–120.2)	−23.3 %	81.58 (49.87–132.8)	23.95 (16.22–36.1)	−70.6 %
Tamil Nadu	Both	23,249.5 (16,644.4-32,655.6)	15,382.8 (11,061.1-21,180.3)	−33.8 %	50.03 (34.94–70.74)	16.07 (11.53–22.03)	−67.9 %
	Female	8181.8 (4600.8-13,694.3)	5939.1 (3123.4-10,480.8)	−27.4 %	35.73 (19.45–60.34)	12.08 (6.42–21.48)	−66.2 %
	Male	15,067.8 (9595.3-23,262.6)	9443.7 (6560.3-12,763.9)	−37.3 %	63.4 (38.67–101.65)	20.26 (14.05–27.55)	−68 %
Telangana	Both	15,517.5 (10,706.6-23,241.9)	11,306.3 (7253-16,501.3)	−27.1 %	81.35 (54.78–125.45)	28.48 (18.19–42.21)	−65 %
	Female	6120.1 (3457.2-9655.2)	4764.1 (2412.0-8553.4)	−22.2 %	65.1 (36.46–105.28)	23.3 (11.74–42.21)	−64.2 %
	Male	9397.3 (5607.0-16,223.1)	6542.2 (4229.3-9753.6)	−30.4 %	97.14 (56.23–174.38)	34.13 (21.69–51.45)	−64.9 %
Tripura	Both	1280.1 (865.8–1967.7)	1032 (718.8–1486.3)	−19.4 %	72.48 (47.18–116.47)	25.07(17.29–36.56)	−65.4 %
	Female	199.9 (114.8–329.3)	162.5 (87.5–314.2)	−18.7 %	22.22 (12.48–38.25)	7.66 (4.12–15.06)	−65.5 %
	Male	1080.3 (694.5–1767.2)	869.5 (589.5–1281.7)	−19.5 %	117.94 (72.87–198.57)	42.44 (28.25–62.58)	−64 %
Union Territories other than Delhi	Both	384.4 (281.1–544)	483.4 (337.3–680.3)	25.8 %	26.53 (18.68–39.19)	11.64 (8.03–16.52)	−56.1 %
	Female	111.8 (62.8–189)	146.5 (82.0–265)	31 %	16.75 (8.83–29.97)	7.26 (4.00–13.28)	−56.7 %
	Male	272.6 (187.2–409.9)	336.9 (231.3–479.7)	23.6 %	35.29 (22.94–55.86)	15.88 (10.57–22.85)	−55 %
Uttar Pradesh	Both	106,727.9 (69,382.7-195,307.7)	144,131.4 (94,815.4-318,723.3)	35 %	121.19 (76.43–236.64)	74.44 (48.14–169.85)	−38.6 %
	Female	15,622.4 (7984.7-26,048.2)	22,147 (10,197.5-40,732.7)	41.8 %	38.13 (18.89–65.83)	22.93 (10.4–42.58)	−39.9 %
	Male	91,105.5 (56,066.8-181,704.8)	121,984.4 (74,227.2-299,463)	33.9 %	190.83 (114.7–409.86)	125.38 (74.96–322.94)	−34.3 %
Uttarakhand	Both	6855.7 (4422.4-10,074.4)	7747.4 (4779.9-11,493.6)	13 %	157.06 (99.57–242.61)	68.35 (41.38–102.26)	−56.5 %
	Female	1811.7 (892.2–3124.1)	1932.6 (863.7–3949)	6.7 %	81.23 (39.41–139.64)	32.98 (14.73–67.2)	−59.4 %
	Male	5044.1 (2994.0-7855.8)	5814.9 (3631.3-8665.4)	15.3 %	234.58 (134.14–379.7)	107.03 (65.08–162.35)	−54.4 %
West Bengal	Both	25,215.7 (17,080.1-39,368.8)	19,385.2 (13,804.8-31,020)	−23.1 %	55.76 (35.79–92.53)	18.21 (12.78–29.63)	−67.3 %
	Female	3726.4 (2153.5-5865)	3232.3 (1925.4-5717.9)	−13.3 %	15.82 (8.84–25.74)	6.07 (3.57–10.74)	−61.6 %
	Male	21,489.3 (13,796.7-36,449.3)	16,152.9 (10,764.7-27,775.1)	−24.8 %	91.64 (54.79–162.04)	29.75 (19.94–51.03)	−67.5 %

Note: ASDR, age-standardized DALYs rates; DALYs, disability-adjusted life-years.

**Fig. 1:**
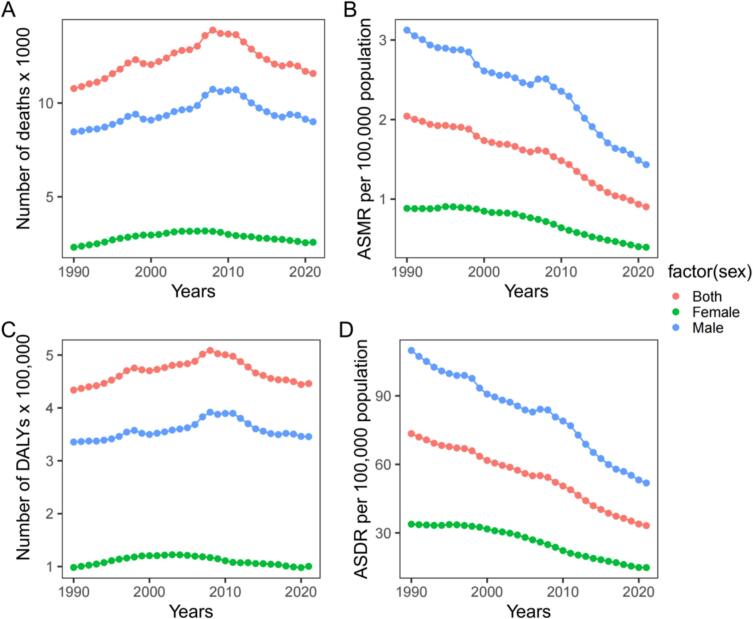
Temporal trends of asthma attributable to occupational asthma. Number of deaths (A), ASMR (B), number of DALYs (C), and ASDR (D), among individuals aged 15 and older in India, 1990–2021. ASMR, age-standardized mortality rate; DALYs, disability-adjusted life years; ASDR, age-standardized DALYs rate.

From 1990 to 2021, the total deaths and DALYs due to occupational asthma among males and both sexes combined showed an upward trend until 2008, after which they gradually declined, while the trend for females remained relatively stable. Male figures were significantly higher than those for females. ASMR and ASDR showed steady declines for males, females, and both sexes (Fig. 1).

### 3.2 The burden of occupational asthma in India states

**Fig. 2:**
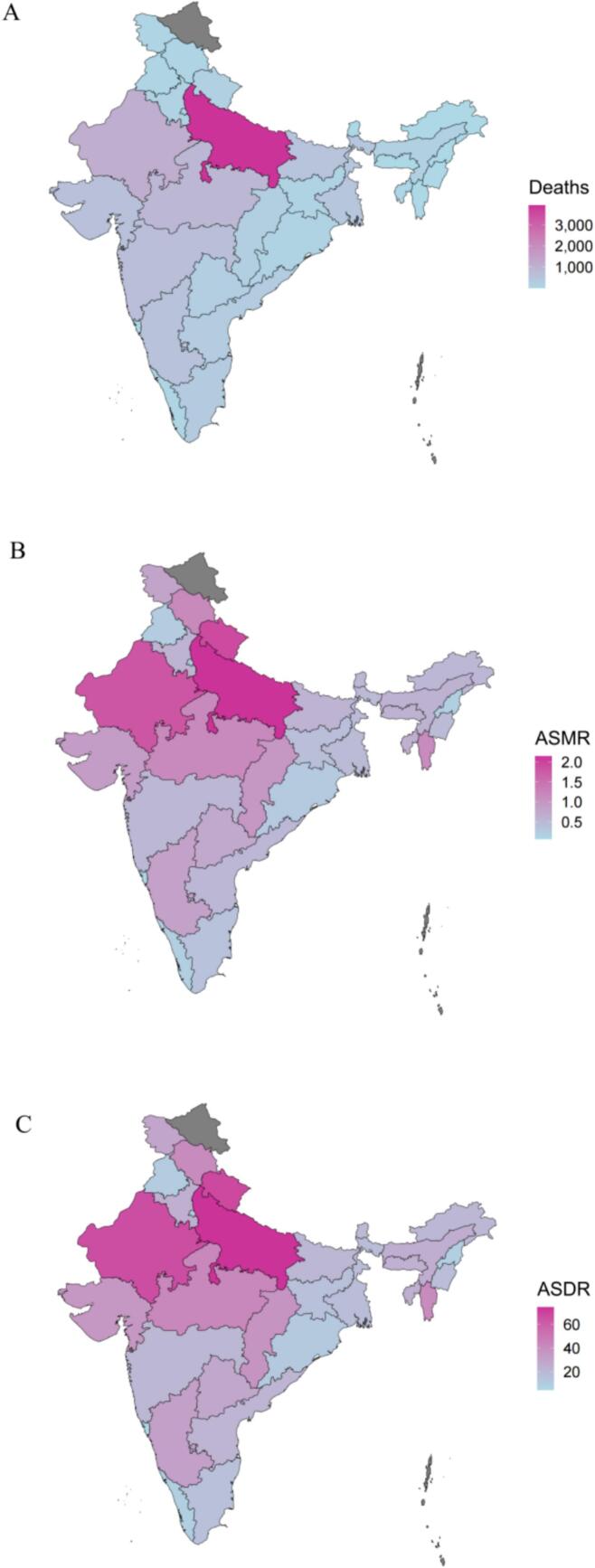
The asthma attributable to occupational asthma burden in terms of deaths (A), ASMR (B) and ASDR (C), among individuals aged 15 and older in India, 2021. ASMR = age-standardized mortality rate; ASDR = age-standardized DALYs rate; DALY = disability-adjusted life year.

In 2021, Uttar Pradesh had the highest number of deaths at 3925.8 (95 % uncertainty interval: 2357.9–9866.2), followed by Rajasthan with 1172.7 (95 % uncertainty interval: 707.1–2034.5), while Goa reported the lowest at 1.6 (95 % uncertainty interval: 1.1–2.3). Compared to 1990, Meghalaya saw the largest increase in deaths at 41.2 %, while Kerala had the largest decrease at −49.6 %. Among males, Uttar Pradesh recorded the highest number of deaths at 3324.2 (95 % uncertainty interval: 1813–9557.5), and Goa the lowest at 1.2 (95 % uncertainty interval: 0.7–1.8). From 1990 to 2021, Uttar Pradesh saw a 39 % increase in male deaths, while Kerala experienced the largest decrease at −56.7 %. For females, Uttar Pradesh reported the highest death toll at 601.6 (95 % uncertainty interval: 245.6–1187.3), and Goa the lowest at 0.4 (95 % uncertainty interval: 0.2–0.8). Manipur saw the highest increase in female deaths at 90.6 %, while Bihar experienced the greatest decrease at −33.4 % (Table 1 and Fig. 2A).

The highest ASMR in 2021 was found in Uttar Pradesh at 2.14 (95 % uncertainty interval: 1.25–5.42), while the lowest was in Goa at 0.08 (95 % uncertainty interval: 0.05–0.11). All states reported decreases in ASMR from 1990, with the largest reduction in Jharkhand at −79.1 %. Among males, Uttar Pradesh had the highest ASMR at 3.63 (95 % uncertainty interval: 1.94–10.55), followed by Rajasthan at 3.04 (95 % uncertainty interval: 1.69–5.53), with Jharkhand experiencing the largest reduction at 85.3 %. In females, the highest ASMR was in Uttarakhand at 0.93 (95 % uncertainty interval: 0.37–1.93), while the lowest was in Delhi and Goa, both at 0.04 (95 % uncertainty interval: 0.02–0.07). Bihar had the largest decrease among females at −71.9 % (Table 1 and Fig. 2B).

In 2021, Uttar Pradesh reported the highest ASDR at 74.44 (95 % uncertainty interval: 48.14–169.85), followed by Uttarakhand at 68.35 (95 % uncertainty interval: 41.38–102.26), with Jharkhand showing the largest decline at −77.7 %. For males, Uttar Pradesh had the highest ASDR at 125.38 (95 % uncertainty interval: 74.96–322.94), and Goa the lowest at 7.24 (95 % uncertainty interval: 5.11–9.85), with Kerala experiencing the largest reduction at −74.9 %. Among females, the highest ASDR was in Uttarakhand at 32.98 (95 % uncertainty interval: 14.73–67.2), followed by Mizoram at 31.55 (95 % uncertainty interval: 16.11–55.99). Bihar showed the largest decrease at −72.3 % compared to 1990 (Table 2 and Fig. 2C).

### 3.3 Age groups

**Fig. 3:**
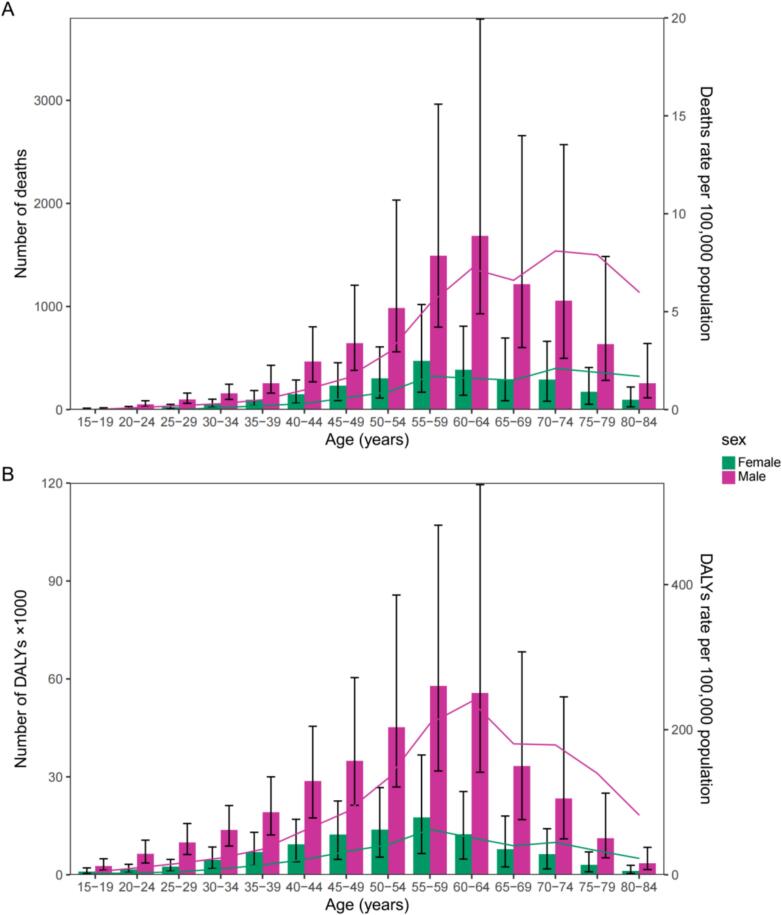
Age distribution of the burden of occupational asthma by sex, in terms of deaths (A) and DALYs (B), among individuals aged 15 and older in India, 2021. DALYs, disability-adjusted life-years.

In 2021, across all age groups, male deaths, DALYs, death rates, and DALY rates were higher than those for females, with little variation among females across age groups. Among males, the number of deaths increased steadily from ages 15–19 to 60–64, after which it gradually declined, with a slight dip in death rates after 60–64, peaking again at 70–74 before declining further (Fig. 3A). DALYs peaked at ages 55–59 and then decreased gradually. DALY rates peaked at ages 60–64 and then declined thereafter (Fig. 3B).

**Fig. 4:**
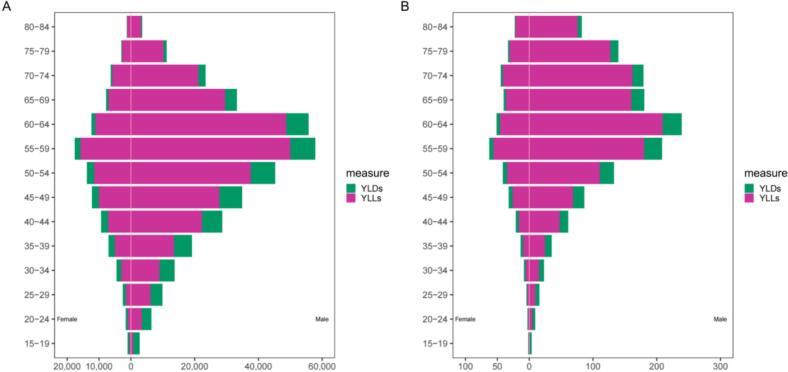
Age distribution of occupational asthma by sex among individuals aged 15 and older in India: Number of DALYs (A) and DALY rates (B), 2021.DALY = YLLs +YLDs; DALY, disability-adjusted life years; YLLs, year of life lost due to premature death; YLDs, years lived with disability.

In 2021, years of life lost (YLLs) represented the dominant burden for both males and females. YLL counts for both sexes began to rise at ages 15–19, peaked at ages 55–59, and then gradually declined. Years lived with disability (YLD) counts showed a similar trend, though YLD counts were significantly lower among females compared to males. Among males, YLL rates began to rise from ages 15–19, peaked at ages 60–64, and then gradually decreased, while female YLL rates remained stable across age groups. YLD rates were consistently low among females. In contrast, YLD rates among males followed a trend similar to male YLL rates (Fig. 4).

## 4 Discussion

From 1990 to 2021, the burden of occupational asthma in India has shown complex trends. While the total number of deaths due to occupational asthma increased by 7.5 %, ASMR and ASDR for both males and females decreased significantly, by around 55 %. This suggests improvements in healthcare, better management of asthma symptoms, or greater awareness of occupational health risks. According to World Health Organization, the major challenges facing asthma in India are inadequate diagnosis and treatment (The Global Asthma Report, 2022). Inhaled corticosteroids are crucial for controlling asthma and reducing long-term complications (O'Byrne et al., 2019), yet their use is severely limited in India, with less than one-third of asthma patients using inhaled corticosteroids (Salvi et al., 2022). The continued rise in absolute mortality reflects the persistent presence of occupational exposures causing asthma, with the highest exposure rates among professional workers and agricultural, hunting, and forestry personnel (Cohen et al., 2023). Geographic location may also be a risk factor for occupational asthma, as sub-Saharan Africa and South Asia are identified as high-risk regions. In the United Kingdom, occupational asthma has been associated with exposure to flour dust or isocyanates, while in France, common causative agents include flour, quaternary ammonium compounds, and isocyanates (; Reeb-Whitaker et al., 2022). These findings highlight the need for further studies on treatment practices and specific exposure causes among India's occupational asthma patients.

There are significant differences in the burden of occupational asthma across various Indian states. Uttar Pradesh, one of India's most populous states, carries the heaviest burden, with ASMR and ASDR figures significantly higher than in other states. This may be due to the predominantly rural population, where agriculture is a key industry. Additionally, the textile and sugar industries are long-standing in Uttar Pradesh, employing a substantial number of workers. India is also the largest producer and consumer of spices, which contain multiple potential allergens that can lead to occupational asthma (Reeb-Whitaker et al., 2022; Upadhyay et al., 2019), and Uttar Pradesh grows a variety of spices. In contrast, Goa reports the lowest burden, possibly due to its small size and low population density. Although studies showing a positive correlation between green spaces and childhood asthma prevalence (Malamardi et al., 2022), Goa's forest cover is high at 56 %, and tourism, rather than industry, is its main sector. In summary, these state-level differences highlight the need for tailored interventions to alleviate the burden of occupational asthma in different regions.

Data show that males carry a disproportionately higher burden of occupational asthma than females, with male mortality rates significantly higher than those of females. This disparity likely reflects sex-specific occupational distribution in India, where men are more commonly employed in high-risk industries (Mannetje et al., 2009). Even within the same occupations, men and women face different specific risk factors. For example, men working as machine operators and in industries like mining, construction, manufacturing, and transportation have a heightened risk of asthma (Agrawal et al., 2014). Additionally, male workers in these sectors may encounter higher concentrations of occupational agents and may lack adequate ventilation or personal protective equipment (Blouin and Lemière, 2024). Addressing these sex disparities requires policies focused on high-risk industries and protective measures for male workers in these environments.

An analysis of age-related differences shows that mortality and disability rates peak in the 55–59 age group. This pattern likely reflects the cumulative effects of long-term occupational exposure, leading to chronic respiratory conditions such as asthma as workers age. The decline in mortality and disability rates after age 65 may indicate that individuals with severe disease may die earlier, or that retired individuals have reduced exposure, resulting in fewer new cases among older age groups. However, the role of population ageing cannot be overlooked. As India's population ages, older individuals may experience longer cumulative exposure to occupational hazards, such as dust, chemicals, and fumes, which could increase their risk of developing severe occupational asthma or exacerbating existing respiratory conditions. Additionally, ageing is often associated with reduced lung function and increased comorbidities, which may further elevate the risk of occupational asthma-related deaths. This emphasizes the importance of early intervention and ongoing health monitoring for workers across all age groups to prevent the progression of occupational asthma and improve quality of life as these workers age. Future studies should explore the interaction between ageing, occupational exposures, and occupational asthma burden in greater depth to better understand the evolving burden of occupational asthma in ageing populations and inform targeted interventions for older workers.

Our study has some limitations. First, the lack of granular data on subnational trends and specific occupational risk factors restricts our ability to identify localized patterns or evaluate policy impacts. Second, data from the Ladakh region are unavailable, potentially introducing geographic bias. Third, we did not assess the prevalence or incidence of occupational asthma, limiting our understanding of non-fatal outcomes. Fourth, we could not evaluate specific occupational exposures (e.g., dust, chemicals) by state or industry, hindering targeted interventions. Although the GBD 2021 framework adjusts for diagnostic improvements, regional disparities in healthcare access and underreporting of occupational asthma may still lead to conservative burden estimates with wide uncertainty intervals. Additionally, our analysis did not incorporate socioeconomic factors or occupational sectors, which may influence occupational asthma burden. Future studies should focus on collecting industry-specific exposure data and refining diagnostic trends to better understand and address the burden of occupational asthma in India. Despite these limitations, our findings provide valuable insights and highlight the need for improved data collection and targeted research.

## 5 Conclusion

This study underscores the persistent burden of occupational asthma in India, with notable variations by sex, state, and age group. While standardized incidence rates have declined, occupational asthma remains a major public health challenge, especially in Uttar Pradesh and among males in high-risk occupations. Addressing these disparities will require targeted occupational health policies, improved monitoring systems, and workplace interventions. Focusing on high-risk populations can help reduce the future burden of occupational asthma and enhance the overall well-being of India's workforce.

## Funding

Not applicable.

## CRediT authorship contribution statement

**Li Wei:** Writing – original draft, Visualization, Methodology. **Xiaoling Liu:** Writing – original draft, Visualization, Formal analysis, Data curation. **Junhang Zhang:** Visualization, Supervision, Data curation. **Donglei Shi:** Writing – review & editing, Supervision, Methodology. **Zhaojun Wang:** Writing – review & editing, Project administration, Conceptualization.

## Declaration of competing interest

The authors declare that they have no known competing financial interests or personal relationships that could have appeared to influence the work reported in this paper.

## Data Availability

The datasets presented in this study can be found in online database. The names of the database can be found below: http://ghdx.healthdata.org/gbd-results.

## Appendix A Supplementary data

Supplementary material 1: Number of asthma-related deaths attributable to occupational asthma in 2021 among individuals aged 15 and older across various states in India. Image 1

Supplementary material 2: Age-standardized mortality rate for asthma attributable to occupational asthmagens in 2021 across various states in India. Image 2

Supplementary material 3: Age-standardized DALYs rate for asthma attributable to occupational asthmagens in 2021 across various states in India. Image 3
